# Digestive problems in rabbit production: moving in the wrong direction?

**DOI:** 10.3389/fvets.2024.1354651

**Published:** 2024-02-07

**Authors:** Malou van der Sluis, Yvonne R. A. van Zeeland, Karel H. de Greef

**Affiliations:** ^1^Wageningen Livestock Research, Wageningen University & Research, Wageningen, Netherlands; ^2^Division of Zoological Medicine, Department of Clinical Sciences, Faculty of Veterinary Medicine, Utrecht University, Utrecht, Netherlands

**Keywords:** *Oryctolagus cuniculus*, gastrointestinal disease, epizootic rabbit enteropathy, dysbiosis, *fusus coli*, fiber, nutrition

## Abstract

Digestive problems, both those with a clear pathogenic origin (e.g., *Escherichia coli*) and those without obvious pathogen involvement [e.g., syndromes like epizootic rabbit enteropathy (ERE)], are common in production rabbits and account for the majority of losses in meat rabbit production. A multitude of nutritional, genetic and housing factors have been found to play a role in the occurrence of digestive problems. However, the exact early pathophysiological mechanism, including the links between aforementioned risk factors and subsequent development and expression of gastrointestinal disease, is less clear, especially in non-specific enteropathies without obvious pathogen involvement. In this review, we aim to shed more light on the derailment of the normal gastrointestinal functioning in rabbits. We discuss a conceptual integrated view of this derailment, based on an “overload” pathway and a “chymus jam” pathway, which may occur simultaneously and interact. The “overload” pathway centers around exposure to excess amounts of easily fermentable substrate (e.g., starch and protein) that might be incompletely digested prior to entering the caecum. Once there, hyperfermentation may result in changes in caecal pH and inhibition of the normal microflora. The second pathway centers around a chymus jam resulting from a compromised passage rate. Here, reduced hindgut motility (e.g., resulting from stress or limited fiber supply) leads to reduced flow of digesta and increased caecal retention times, which might lead to the production of abnormal caecal fermentation products and subsequent inhibition of the normal microflora. A central role in the presumed mechanism is attributed to the *fusus coli*. We discuss the suggested mechanisms behind both pathways, as well as the empirical substantiation and alignment between theoretical concepts and observations in practice. The proposed hypotheses may explain the effect of time-based restriction to prevent ERE, which is widely applied in practice but to date not really understood, and suggest that the particle size of fiber may be a key point in the normal functioning of the colon and *fusus coli*. Further insight into the circumstances leading to the derailment of physiological processes in the rabbit hindgut could provide a meaningful starting point to help improve their gastrointestinal resilience.

## 1 Introduction

Rabbits (*Oryctolagus cuniculus*) are herbivorous hindgut fermenters that are adapted to digesting high fiber diets consisting largely of grass (1, 2). Their gastrointestinal tract is complex and the digestive process is sensitive to disruption and subsequent gastrointestinal disease (1–3). Indeed, gastrointestinal disease is common in both pet and production rabbits, and was found to be the primary cause of death of meat rabbits, accounting for approximately two thirds of the mortalities on rabbit farms in Switzerland (4). The gastrointestinal problems that are seen in meat rabbits can be divided into two main types: those with a clear pathogenic origin [e.g., *Escherichia coli*; (5)] and those without a clearly identifiable pathogen involvement [e.g., syndromes like epizootic rabbit enteropathy (ERE); (6)] that are understood less well.

Under normal conditions, rabbits have a high feed intake [65–80 grams per kilogram body weight; (7)] and a high metabolic rate, and the food passes through the gut rapidly (2, 8). In this process, indigestible fiber is quickly eliminated from the digestive tract (2). Caecotrophy helps to complete the digestion of (high-fiber) plant-based components, facilitate assimilation of proteins and other nutrients that are synthesized by caecal bacteria, and maintain gut bacterial populations (1). The caecal microflora plays a large role in rabbit health (3), as an active symbiotic microflora is considered to help prevent overgrowth of a pathogenic microflora (9). While alterations in gut microbiota might be a primary cause of digestive pathologies in rabbits, feeding a nutritionally balanced diet can aid in the prevention of digestive disorders through two main mechanisms (10). First, balanced diets can promote reduced retention times of digesta in the digestive tract (10), as long retention times might otherwise contribute to a destabilization of the caecal microbial activity and digestive problems (11). Second, balanced diets may reduce the flow of easily available substrates into the fermentative area (10) where these easily fermentable substrates may initiate dysbiosis (12). In addition to a high fiber content positively affecting gastrointestinal health [reviewed by Gidenne (13)], high levels of easily fermentable substrates in the diet (e.g., starch and protein) negatively affect rabbit gastrointestinal health (Table 1). Apart from feed composition, also fiber particle size and feed restriction (quantitative and in time) seem to affect the occurrence of digestive problems or general mortality in production rabbits (Table 1). However, it is not yet clear why (time-based) feed restriction has clinically yielded positive effects [e.g., (11, 20, 28)]. Additionally, genetic, housing and sanitation, and stress-related factors can contribute to the occurrence of digestive problems and associated mortality in production rabbits [Table 1; (3)]. These findings demonstrate the complex and specialized nature of the rabbit gastrointestinal tract as well as its sensitivity to disturbances by external factors, especially nutrition, which lead to onset of digestive problems.

**Table 1:** Overview of some of the main factors that have been linked to the occurrence of digestive problems or (general) mortality in meat rabbits, with references to examples of studies.

**Factor**	**Observations**	**References**
Feeding	Increased dietary digestible fiber is linked to lower mortality after weaning or fewer digestive disturbances^a^	(9, 14)
	Increased dietary acid detergent fiber is linked to lower mortality after weaning^a^	(15, 16)
	Fiber particle size might be linked to mortality differences	(17)^b^
	Increased dietary starch is linked to increased mortality after weaning^a^	(18)
	Lower dietary crude protein levels are linked to reduced mortality after weaning^a^	(19)
	Feed restriction is linked to lower mortality after weaning	(20, 21)
Genetics	There is genetic variability in resistance to digestive problems	(22, 23)
Housing and sanitation	Larger group sizes are linked to increased general mortality	(24)
	Housing type might affect overall mortality rates, with inconsistent observations across studies	Dal Bosco et al. (25) for example observed lower mortality in cages than in pens (but group sizes differed too), while others [e.g., (26)] observed no differences between cages and pens.
	Floor type might affect overall mortality rates, with lower mortality with wire net floors	(25)
	Sanitary factors are linked to differences in general mortality, with lower general mortality when cleaning and disinfection take place between cycles	(27)

a It is important to keep in mind that the effects of different feed component levels in a diet may be difficult to disentangle, as a change in the level of one component per definition changes relative levels of other feed components.

b Sobri et al. (17) studied this in interaction with NDF levels and exact fiber particle sizes were not given.

Harcourt-Brown (3) provides an extensive overview of pathological mechanisms that take place during digestive problems. However, the exact pathophysiology and mechanisms responsible for initiating the chain of events leading to altered gastrointestinal functioning has yet to be unraveled. In the literature, empirical substantiation seems to be limited and incomplete but several hypotheses and suggestions have been postulated to indicate what might be happening, and are discussed further on. Nevertheless, an integrated view of the (actual initiation of the) derailment process, which could have great value as a starting point for further research on—and future prevention of—gastrointestinal problems in rabbits, is currently lacking. The aim of this paper is therefore to expand upon the currently limited concepts that exist on the early—especially nutrition induced—development of digestive disorders, and provide a conceptual framework that integrates current knowledge regarding the gastrointestinal derailment in rabbits with a focus on the non-specific enteropathies (e.g., ERE). To gain insight into the derailment process of the gastrointestinal tract, we studied scientific literature on gastrointestinal health, disease and risk factors. The work in this paper is a spin-off of a larger project on improving gastrointestinal resilience in rabbits, and no formal systematic review was performed. Instead, multiple separate literature searches were performed for different aspects of gastrointestinal health or functioning in rabbits, mainly using Scopus, Google Scholar and Google searches and a snowball approach. Following an overview of current knowledge on normal functioning of the gastrointestinal tract in rabbits, we will elaborate on two new conceptual and possibly interrelated and concurrent pathways of derailment, notably an “overload” pathway and a “chymus jam” pathway, and evaluate to what extent these align with observations from practice. While new in their terminology and integrative approach, these concepts are based upon and inspired by scientific work of others, as will be discussed in the respective sections further below.

## 2 Normal gastrointestinal functioning

**Figure 1:**
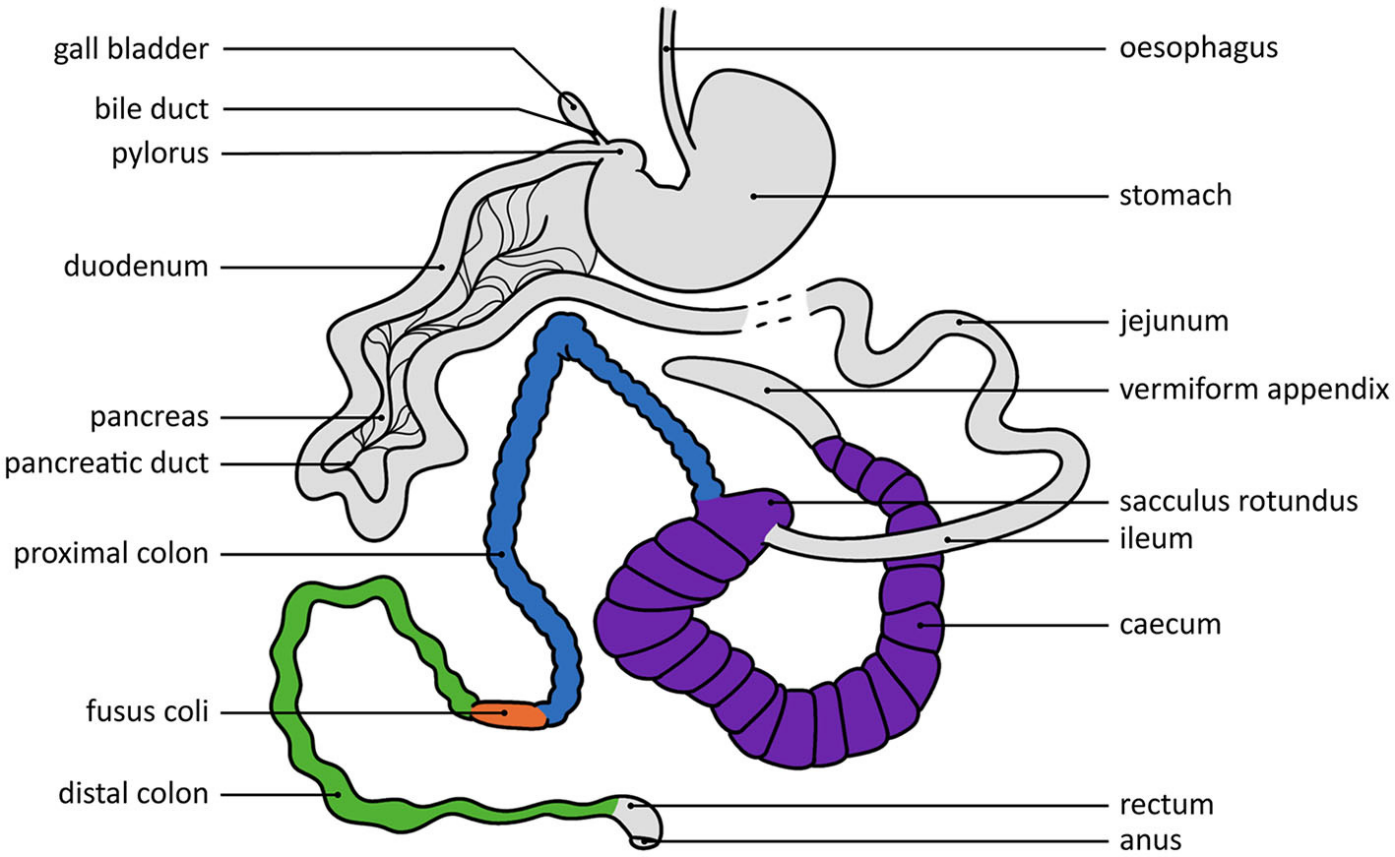
Schematic view of the rabbit gastrointestinal tract. The structures that play a key role in the hypothesized pathways are highlighted in different colors. The dashed line indicates a continuation of this structure that is not fully shown here. Visualization by Mandy Beekmans, adapted from Cheeke (12), Redback Rabbit Boarding (29), and Weebly (30).

An extensive review of the anatomy and histology of the rabbit gastrointestinal tract is beyond the scope of this review, but is described elsewhere [e.g., (3, 8, 12)]. Here, we focus on the physiological aspects and functioning of the digestive system. A schematic visualization of the gastrointestinal tract of rabbits is shown in Figure 1. Rabbits have a highly complex gastrointestinal tract, that makes up around 10–20% of their body weight (2). The stomach is simple and thin-walled, and serves as a reservoir for ingesta, containing food, caecal pellets and hair that has been ingested, and is virtually never empty (2, 31). In adult rabbits, the stomach pH is low (pH 1–2) during ingestion of food, which results in the food being effectively sterilized (2, 8, 32). The digesta normally reside ~3–6 h in the stomach, and are then gradually passed into the small intestine through short bursts of strong stomach contractions (33).

The digesta entering the small intestine are diluted by bile, the first intestinal secretions and pancreatic juice (33). The digesta reside in the small intestine for ~1½ h, and in the duodenum and jejunum most of the digestion of carbohydrates and simple proteins takes place (8, 33). The digested monosaccharides and amino acids, volatile fatty acids (VFAs), vitamins, and digested microbial organisms are absorbed through the jejunal brush border (8). Any particles that are not broken down in the small intestine move into the caecum and colon (12, 33).

The caecum of a rabbit is large and serves as an anaerobic fermentation chamber, where the ingesta, together with mucopolysaccharides secreted from the mucosa, constitute an important carbohydrate source for caecal fermentation (8, 31). The microbial flora in the caecum contributes to the breakdown of ammonia (NH_3_), urea, proteins, cellulose, and enzymes from the small intestine (8). The resulting products are the protein and enzyme structures of the microbiota itself, that are obtained later through the process of caecotrophy, and by-products in the form of VFAs that are absorbed through the caecal and colonic walls (8). After 2–12 h in the caecum, the remaining caecal contents, consisting of undigested food particles and caecal bacteria, move into the colon (33).

The rabbit colon consists of three different parts: a proximal colon, followed by a muscular thickening (the *fusus coli*), and the distal colon (8). The *fusus coli* is a distinct part of the colon that is ~4 cm long and—unlike other parts of the colon—has no taeniae or haustra, and relatively few microvilli, but mostly longitudinal folds with deep furrows or crevices that promote reabsorption of water, potassium and sodium and drying out of the intestinal contents (34). Physiologically, the *fusus coli* furthermore serves as a pacemaker for haustral, segmental and peristaltic contractions (8, 35–38), thereby regulating colonic motility. The *fusus coli* is therefore considered to be vital for normal gastrointestinal functioning as it aids (via colonic motility) in the simultaneous separation of indigestible fiber (that is eliminated in the form of hard pellets) and digestible components (that are moved backwards to the caecum for further fermentation through antiperistalsis) based on particle density and size (2, 8, 12, 35, 37).

What happens next to the digestible components depends on the timing of the caecal contents entering the proximal colon. Every morning, but sometimes more often (39), few biochemical changes occur and soft pellets are formed from the caecal content that is expelled into the colon (33). These soft pellets are excreted from the anus and directly ingested by the rabbit through caecotrophy, after which they undergo the entire digestive process again, some components potentially for multiple times in a row (33). If the caecal content enters the colon at a different time, the contractions result in the earlier-mentioned separation of digesta contents to be sent back and the solid part to be passed on, resulting in hard pellets, consisting of mainly large particles, that are excreted (33).

## 3 Pathways of derailment

Several factors can affect gastrointestinal health in rabbits, including stress, feed composition and distribution, genetic factors, and housing and sanitation factors (Table 1). The autonomic nervous system and adrenal glands are involved in regulating the activity of the *fusus coli* (8) whereby hypersecretion of adrenalin (associated with stress) results in a slowing down of the digestive activity, posing a risk factor for digestive problems (33). Feeding strategies have been suggested to affect gastrointestinal health through competitive exclusion among bacteria, leading to non-pathogenic species predominating pathogenic ones (40). Generally, an active caecal symbiotic microflora is presumed to help prevent overgrowth of a pathogenic flora (9). Furthermore, feeding strategies could help to promote the development of intestinal barrier mechanisms (40). Several authors suggest that dietary or nutritional imbalances may not be the root cause of digestive health problems [e.g., (11, 19)] but rather increase susceptibility to digestive disease (11), potentially by altering gut microbiota, which is hypothesized to be a primary cause of digestive problems (10). Two conceptual pathways may explain the onset of derailment and microbial alterations, that is, the so-called “overload” pathway and “chymus jam” pathway.

### 3.1 The “overload” pathway

**Figure 2:**
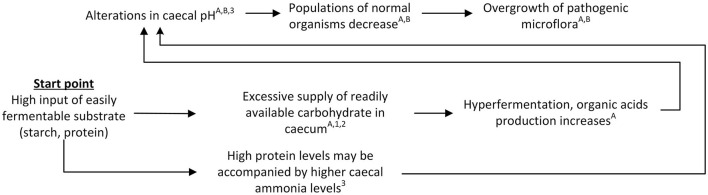
Schematic view of the “overload” pathway leading to gastrointestinal problems in rabbits. References for suggested pathways indicated with letters, empirical work indicated with numbers. ^A^(12); ^B^(41); ^1^(18); ^2^(15); ^3^(42).

The first pathway that may lead to a derailment of gastrointestinal processes involves an overload of the caecum with easily fermentable substrates such as starch or protein (see Figure 2).

The suggested mechanism underlying the “overload” pathway is inspired by and collated from several observations and findings that have been previously published in the literature. Under normal circumstances, most of the digestion of simple proteins is reported to take place in the duodenum and jejunum (8). Starch is also mainly digested in the small intestine, but any remaining starch entering the caeco-colic segment may be quickly hydrolysed and fermented there (43). In case of an overload of fermentable carbohydrate, excess starch might be transferred to the caecum, supposedly negatively affecting the fibrolytic bacteria (44) and leading to hyperfermentation and lowering of the caecal pH due to increased production of organic acids (12, 41). This, in turn, could allow proliferation of undesirable microflora that are normally only present in small numbers (12, 41). Similarly, excess dietary protein may negatively affect the caecal environment and microbiota as digestion of excess protein that enters the caecum could lead to increased caecal NH_3_ and pH levels that may stimulate proliferation of pathogens [Morisse et al. (45) in Cheeke (12)].

In terms of empirical evidence for this “overload” pathway, several studies have shown that high starch levels may lead to increased mortality or incidence of digestive problems in young rabbits post-weaning (18, 46). Blas et al. (18) studied rabbits that were either fed a diet consisting of 16.4% starch and 15.3% crude fiber, or a diet consisting of 24.8% starch and 11.6% crude fiber. Rabbits fed the second high-starch diet were observed to have a higher mortality between 28 to 49 days of age compared to rabbits fed the low-starch diet. In addition, the starch content in the ileal digesta was higher in rabbits fed the high-starch diet compared to rabbits fed the low-starch diet, and there was a statistical trend for a higher caecal digesta starch content. Based on these findings, the authors suggested that the increased mortality for rabbits fed the high-starch diet might have been related to a starch overload in the hind gut and subsequent undesirable fermentation and growth of the caecal microflora (18). Laurent-Bennegadi et al. (46) studied the effects of a diet with a higher starch level and a fiber deficiency compared to a diet with a standard fiber concentration, and found a higher incidence of digestive problems in rabbits fed the higher-starch fiber-deficient diet. When comparing the caecal fermentative profile of healthy rabbits and dying diarrheic rabbits, lower total VFAs were observed in the sick rabbits, with decreased levels of acetate (C_2_) and butyrate (C_4_), but an increase in propionate (C_3_) levels compared to healthy rabbits, resulting in a reverse C_3_:C_4_ ratio in sick rabbits. It was suggested that this might be due to a decrease in available substrates and subsequent modification of microbiota balance. They also observed a significant increase in the minor VFAs isobutyric, valeric, and isovaleric acid, as well as a higher concentration of ammoniacal nitrogen (NH_3_-N) and higher caecal pH, and suggest that their observations could indicate a more proteolytic profile (46). In line with observations from Laurent-Bennegadi et al. (46), Marounek et al. (47) reported that, among other things, more propionate and less acetate was formed from starch than from pectin when examining fermentation patterns and yield of microbial protein in cultures of rabbit caecal contents supplied with glucose, xylose, starch, pectin or xylan, thereby showing that substrate characteristics can affect fermentation patterns. Together, the findings from these studies indicate that high-starch diets lead to higher starch concentrations in the caecum, which in turn results in a fermentation pattern that differs from low-starch diets.

Tazzoli et al. (48), who studied rabbits on diets with different crude protein levels, observed that decreased dietary protein levels resulted in a reduced mortality and an increased caecal pH. Based on their findings, the authors suggested that high caecal protein contents could benefit the entire microbial population, but that protein excess could possibly benefit some of the pathogenic strains to a higher extent (48). Similar to Tazzoli et al. (48), Chamorro et al. (10) observed a lower mortality in rabbits fed lower crude protein levels. In addition, they observed a decreased ileal crude protein flow, a lower frequency of *Clostridium perfringens* in the ileal microbiota, and a higher fundus pH, while no difference was observed in caecal pH (10). Martínez-Vallespín et al. (42) studied effects of diets with variable crude protein, starch, acid detergent fiber (ADF), and neutral detergent soluble fiber (NDSF) levels, and observed, amongst other things, increased caecal digesta pH, and reduced VFA and NH_3_ concentrations in the caecal digesta for rabbits fed lower crude protein levels. Based on their findings, the authors suggested that the reduced VFA concentration and increased pH could indicate a lower microbial activity (42). Overall, the findings from these studies indicate that higher crude protein levels may result in changes in caecal pH, microbiota composition and fermentation product composition.

### 3.2 The “chymus jam” pathway

**Figure 3:**
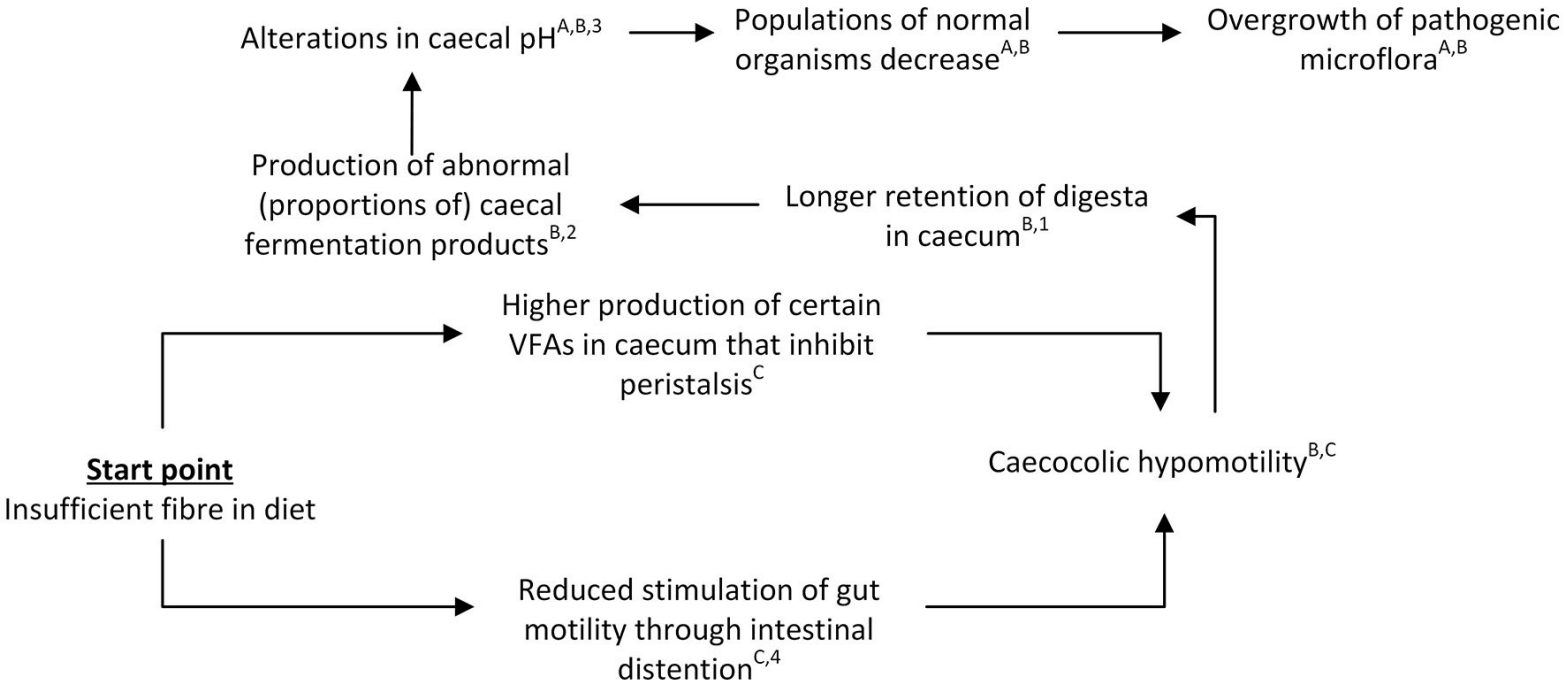
Schematic view of the “chymus jam” pathway leading to gastrointestinal problems in rabbits. References for suggested pathways indicated with letters, empirical work indicated with numbers. ^A^(12); ^B^(41); ^C^(49); ^1^(50); ^2^(15); ^3^(42); ^4^(51).

The second conceptual pathway for the derailment of the gastrointestinal process centers around compromised passage rates and subsequent accumulation of chymus in the caecum and proximal colon, here referred to as the “chymus jam” pathway. This pathway is shown in Figure 3.

The suggested mechanism underlying the “chymus jam” pathway is inspired by and based on different components or sub-mechanisms that have previously been reported in the literature. Central to this pathway is the reduced motility of the hindgut, which may result from stress or a too limited (indigestible) fiber supply (3, 12, 49). Stress and stimulation of the sympathetic nervous system are reported to negatively affect normal functioning of the *fusus coli*, thereby slowing down peristalsis and inhibiting gut motility and digestion (8, 49). In case of shortage of fiber or inadequate structural aspects of the fiber (e.g., particles are too small) retention times in the proximal colon may be increased, as the passage of large particles of indigestible fiber is thought to stimulate gut motility through intestinal distention (49). When the fiber particles are fine ground, this is thought to interfere with the favorable effect of fiber on hindgut motility (12). Reduced gut motility has been suggested to result in longer retention of digesta in the caecum (41). However, we hypothesize that during this period of reduced gut motility the *fusus coli* will continue to send back digesta to the caecum in the absence of large, indigestible (fiber) particles, while the ileum also continues to supply nutrients to the caecum (albeit slower). These processes—together with a lack of clearance of the proximal colon—may subsequently lead to overload and overfilling of the caecum and proximal colon, thus resulting in a “chymus jam” and increased caecal retention times. Prolonged retention of digesta in the caecum may in turn contribute to a destabilization of the caecal microbial activity, thereby favoring digestive problems (11). In terms of the mechanism behind this, Gidenne et al. (11) speculate that the increased retention times and low caecal turnover of digesta may result in an insufficient supply of substrates for the fibrolytic flora, while Oglesbee and Lord (41) suggest reduced caeco-colic motility to induce production of abnormal caecal fermentation products and caecal pH alterations which in turn may lead to inhibition of normal microbiota, and propagation of pathogenic ones. In either case, compromised passage rates are suggested to significantly affect rabbit gastrointestinal health, and ingestion of indigestible fiber in particular appears to be of importance for normal gastrointestinal motility and lower gastrointestinal retention times (8, 12).

In terms of empirical evidence for this “chymus jam” pathway, experimental work using an isolated rabbit distal colon has demonstrated that gut dilation promotes peristalsis (51), suggesting that the presence of digesta in the colon, which causes gut dilation, already promotes peristalsis in itself. In addition, VFA production in the caecum is hypothesized to promote peristalsis. Jehl and Gidenne (9) found higher caecal VFA concentrations (most notably acetate) in rabbits fed a high digestible fiber diet compared to rabbits fed a starch-rich diet. Additionally, Garcia et al. (52) observed that increasing dietary NDF content tended to increase the proportion of acetic acid in the caecum whereas the proportion of butyric acid decreased, while Gidenne et al. (15) reported higher butyrate and lower acetate levels for lower ADF levels in the diet. Increased proportions of butyrates in the caecum in particular have been suggested to inhibit peristalsis (3), thereby negatively affecting the rabbits' gastrointestinal health. However, in rats, the opposite has been reported as butyrate increased colonic motility (53). Hence, despite uncertainty regarding the role of butyrate on colonic motility in rabbits, it is clear that gut dilation promotes peristalsis and that alterations in dietary composition result in alterations in VFA production.

Reduced digestible fiber levels have been shown to increase retention times of digesta in the gastrointestinal tract. For example, Gidenne et al. (15) examined effects of different levels of ADF (where ADF was replaced by starch for some groups), and observed longer mean whole-tract retention times in rabbits fed lower levels of ADF. Similarly, Gidenne and Perez (50) showed that replacing digestible fiber by starch resulted in a slower mean gastrointestinal tract passage rate. Additionally, they observed increased retention times in the caecum for small particles (<0.3 mm), but not for large particles. In addition to the longer retention times in rabbits fed lower ADF levels, Gidenne et al. (15) observed, amongst others, (1) a higher caecal pH, (2) a higher NH_3_ level, (3) lower acetate levels and higher butyrate levels (as previously mentioned), and (4) a higher mortality. Michelland et al. (54) studied effects of a sudden dietary change at 49 days of age, where rabbits were converted from a control diet to a fiber deficient diet, with simultaneous changes in the type of fiber and protein sources due to diet formulation constraints. They observed, compared to rabbits that remained on the control diet, a less acidic, more reductive and drier caecal environment, as well as an increase in NH_3_-N and a decrease in total VFA concentration, modified bacterial community structures, and a lower number of 16S rRNA gene copies of total bacteria. Based on these findings, the authors concluded that a change in dietary fiber level modifies both the quantity and structure of the bacterial community. In line with these findings, Gomez-Conde et al. (55) observed a tendency for lower *C. perfringens* and *Campylobacter* spp. caecal colonization in rabbits fed a high NDSF diet (but similar concentrations of total dietary fiber, starch and crude protein). Summarizing, it has been shown that lower fiber levels are linked to longer retention times in rabbits and, although the exact (direct) effects of longer retention times on fermentation patterns are unclear, also to alterations in fermentation patterns.

### 3.3 The pathways combined

It is difficult to separate effects of higher starch vs. low fiber exposure, as these dietary changes are inextricably linked and paired with one another: a change in the level of one component per definition changes relative levels of other feed components. This means that a change in dietary fiber, for example, may also have an effect on dietary starch and may hereby affect both discussed pathways simultaneously. For that reason, it is important to also examine the potential interaction of the two proposed pathways.

**Figure 4:**
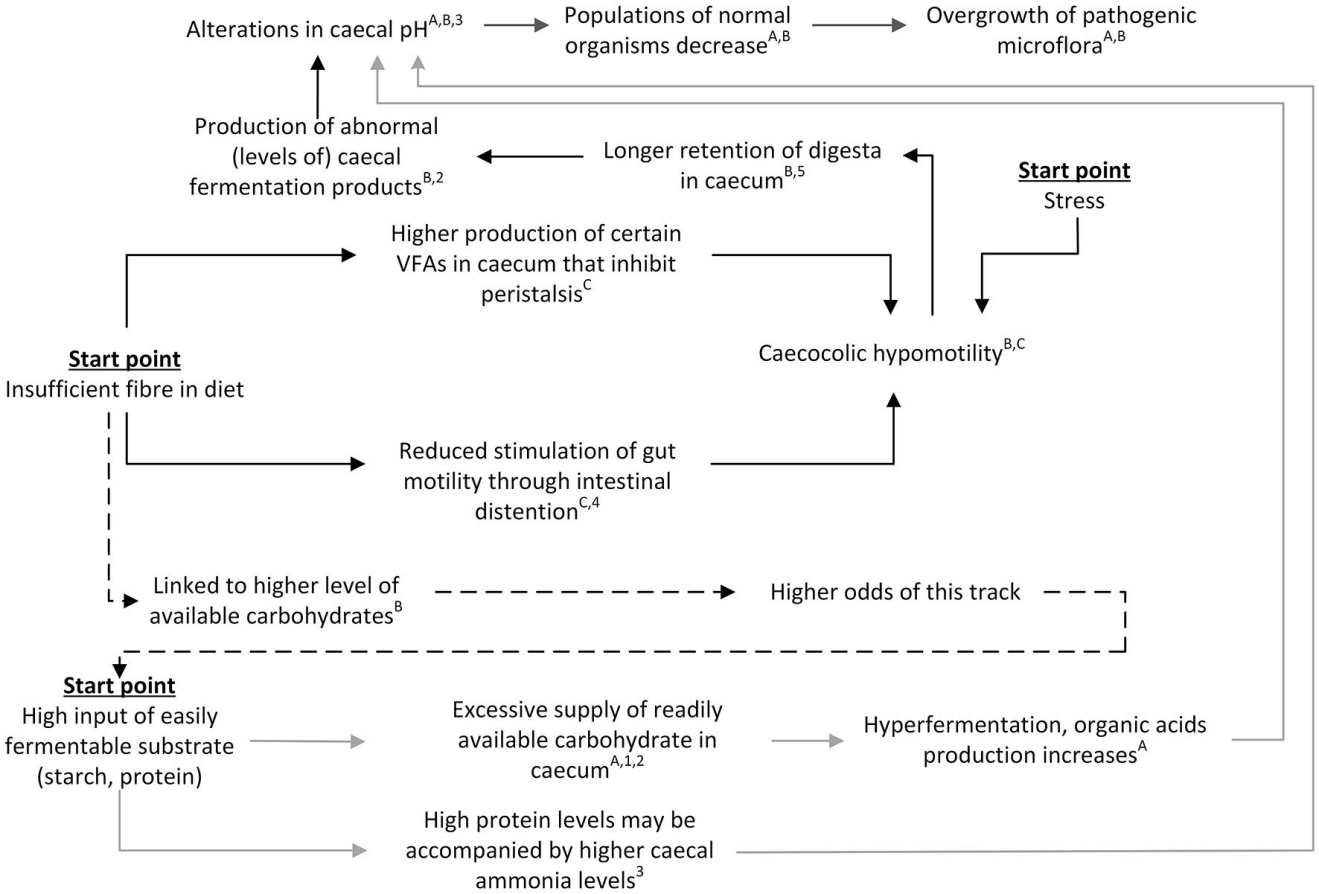
Schematic view of the integrated pathways leading to gastrointestinal problems in rabbits. Gray arrows represent the “overload” pathway, black arrows represent the “chymus jam” pathway. Dotted arrows indicate the indirect link between the two pathways. References for suggested pathways indicated with letters, empirical work indicated with numbers. ^A^(12); ^B^(41); ^C^(49); ^1^(18); ^2^(15); ^3^(42); ^4^(51); ^5^(50).

In Figure 4 we have attempted to schematically visualize how the two pathways would interact and be affected by external influences such as stress and dietary composition. As illustrated in this figure, insufficient fiber, too much easily fermentable substrate and stress may all feed into the same overall derailment process, that ends with an overgrowth of pathogens in the caecum and subsequent pathology such as intestinal gas distention and even death [(41); also in line with the interconnected pathways presented in Figure 10.1 of Harcourt-Brown (3)]. In addition, Figure 4 shows that the pathways and consequences of excess amounts of easily fermentable substrate and shortage of fiber do not necessarily occur in isolation but rather exert concurrent or even additive effects. High fiber diets generally contain lower levels of easily fermentable carbohydrates (41) and as such both decrease the risk of “chymus jam” as well as “overload.” This interdependency of the pathways through diet composition makes it difficult to determine or predict the relative importance of each separate pathway. Indeed, the relative effects of fiber and starch levels are under debate in literature, but there is some evidence for a larger effect of fiber compared to starch. For example, Gidenne et al. (56) studied the ileal flow of fiber and starch using three diets with ADF:starch ratios of 1.9, 0.7, and 0.4, respectively. For all three diets, ileal starch digestibility was high (93.0–98.7%) in adult rabbits, resulting in a low ileal starch concentration (mean 1.3% of dry matter^1^) and rendering starch overflow unlikely as a cause for the observed digestive problems. Nevertheless, the authors did not exclude the possibility of a potential effect of a starch overflow when feeding a lower quality starch or around weaning, due to the lower secretion of pancreatic amylase at that age (56). In line with this, Gidenne et al. (57) compared effects of diets with different starch sources (wheat, barley, maize or extruded maize) and observed no relationship with rabbit mortality due to digestive disorders. These findings suggest that it is more likely that the “chymus jam” pathway plays a larger role than the “overload” pathway. However, it does not necessarily exclude the “overload” pathway from playing a role. Empirical evidence by Martínez-Vallespín et al. (19), who studied effects of different combinations of three dietary adjustments (increased ADF (lower starch), increased NDSF (lower starch), and reduced crude protein levels), showed that the three dietary adjustments combined resulted in the largest reduction in mortality in growing rabbits, also during an ERE outbreak, indicating additive effects.

## 4 Discussion

In this paper, we have presented two conceptual and possibly interrelated and concurrent pathways of derailment, notably an “overload” pathway and a “chymus jam” pathway, that could lead to significant dysfunction of the gastrointestinal tract as observed in rabbits with non-specific enteropathies such as ERE. Below, we will evaluate in more detail to what extent the aforementioned pathways align with observations from practice, as this will be helpful to determine whether these pathways may indeed play a role in the development of gastrointestinal disease in production rabbits.

In Table 1, we highlighted several of the main observations from practice that identify factors that are linked to the occurrence of gastrointestinal problems in rabbits, including (1) positive effects of increased fiber; (2) negative effects of increased starch; (3) positive effects of lower crude protein levels; (4) effects of fiber particle size (not specified); (5) positive effects of feed restriction; (6) genetic predispositions; and (7) effects of housing and sanitary factors. Genetics could affect many regulatory mechanisms in the gastrointestinal tract, while sanitary factors would likely be linked to enteropathies with distinct pathogen involvement (as opposed to the derailment of the gastrointestinal tract without obvious pathogen involvement, as evaluated here). Similarly, housing factors are mostly linked to sanitary aspects [e.g., wire floors may result in lower contamination risks; (58)] or infection pressure [e.g., larger group sizes may increase infection pressure; (59)], or might affect stress levels [e.g., linked to social interactions; (59)]. Hence, these three factors will not be discussed in further detail here.

The positive effects of increased fiber, as reported in numerous studies [e.g., (9, 14–16)], align well with both conceptual pathways, as increased fiber directly reduces the odds of the “chymus jam” pathway derailment (see also section “chymus jam” pathway) and indirectly reduces the odds of the “overload” pathway (Figure 4). Similarly, the negative effects of increased starch [e.g., (18)] and positive effects of lower dietary crude protein levels [e.g., (19)] align well with each conceptual pathway, as both starch and protein feed directly into the “overload” pathway (see also section “overload” pathway) and indirectly into the “chymus jam” pathway (Figure 4).

Interventions aimed at reducing mortality rates by manipulating fiber particle size [e.g., (17)] also align well with the discussed conceptual pathways, as particle size can affect gastric motility due to intestinal distention [(49); Figure 4]. Mechanical separation of the digesta is reported to take place in the colon, where water-soluble substances and fine particles of <0.3 mm in diameter are moved back toward the caecum, and coarse particles of more than 0.3 mm in diameter are moved forward to the distal colon (7). In other words, large fiber particles may stimulate the *fusus coli* to send chymus toward the distal colon, thereby reducing the amount of chymus in the proximal colon. However, it is currently unclear whether the *fusus coli*, during the hard feces phase, only allows digesta to pass to the distal colon when large particles are present, or whether it also allows digesta to (partially) pass if large particles are absent. If only sending digesta to the distal colon when large particles are present, with smaller particles tagging along, a lack of large particles may result in the *fusus coli* repeatedly returning small particles to the caecum, thereby increasing retention times, as smaller particles would only be sent to the distal colon during the soft feces phase. Previous studies have indeed indicated that fiber particle size has an effect on mean gastrointestinal retention times of digesta in rabbits, with (larger proportions of) smaller particles resulting in increased mean ileo-rectal and caecal retention times (60, 61), which could predispose rabbits to overfilling of the upper colon (the “chymus jam” hypothesis; Figure 4). However, if the *fusus coli* sends a substantial proportion of digesta to the colon regardless of whether large particles are present, this hypothesis does not hold as a shortage in large fiber particles would not necessarily result in a higher return of small particles to the caecum. Currently, it is unclear whether large particles play a role in the flow of digesta into the distal colon or not, and how the *fusus coli* affects these processes. Further understanding of the functioning of the *fusus coli* and the flow of particles would be required to gain further insight into the exact pathophysiologic mechanisms underlying gastrointestinal derailment.

The observation that feed restriction, both in volume and in time, is linked to a lower mortality after weaning [e.g., (20, 21, 62)] was an important reason for us in the search for the mechanism behind gastrointestinal derailment in rabbits, but remarkably did not come up directly in relation to the proposed pathways of derailment. The positive effects of feed restriction are poorly understood physiologically [e.g., (11, 20, 28)], and at first glance appear to fit the conceptual pathways poorly. After all, both the conceptual pathways (Figure 4) and literature [e.g., (11)] suggest that anything that increases gastrointestinal retention times will negatively affect gastrointestinal health, and volume-based feed restriction suggestively would increase retention times as reduced food intake would limit intestinal distension, thereby leading to and exacerbating decreased gut motility (41, 49, 51, 63). Therefore, it would logically be assumed that volume-based feed restriction would be detrimental rather than beneficial for gastrointestinal health. However, reduced intake of food could actually reduce the risk of the “chymus jam” and “overload” pathway, as the intake of (easily fermentable) substrates would also be reduced. Time-based feed restriction, during which rabbits only have access to food for part of the day, may also intuitively be expected to increase the risk of digestive problems, as the gastrointestinal tract could become overloaded at times when rabbits have access to the food, especially since some studies have shown that time-based feed-restricted rabbits tend to eat similar amounts, yet in a much shorter time, compared to *ad libitum* fed rabbits (64). Literature and practice, however, show the reverse: commercial growing rabbits are commonly fed in a time-restricted mode, and this intervention whereby food is temporarily withdrawn for 9–12 h substantially reduces the incidence of digestive problems such as ERE [personal communication and practice observations, Rommers and de Greef; (64)]. Night-restricted feeding has been suggested to positively affect gastrointestinal health through improvement of the diurnal rhythm of the gut microbiome (64). However, this contradicts the findings from Martignon et al. (65), who studied rabbits on *ad libitum* vs. restricted diets (75% of *ad libitum*) that were either provided as a single daily meal or distributed across 13 feedings per day. In this study, a statistical trend (*p* = 0.056) for a lower morbidity was observed among the rabbits fed 13 times per day compared to those receiving a single meal per day. An explanation for these different findings may be that the *fusus coli* always lets some of the digesta pass through. If this assumption is accurate, temporary absence of feed intake may present a similar opportunity for the caecum and proximal colon to empty themselves as observed during small portion feeding across the day, as both interventions can help to avoid a chymus jam, regardless of whether there is stimulation from the ingestion of food. Especially for rabbits that are fed high digestible diets with limited fiber, temporary fasting would then allow emptying of the caecum and proximal colon, which could potentially compensate for the lower gut motility and longer retention times resulting from fiber deficiency. Whether the *fusus coli* indeed always lets some of the digesta pass through or whether other mechanisms are in place to explain the beneficial effects of feed restriction therefore deserves further research.

Overall, the conceptual pathways discussed in this paper thus seem to align well with the empirical and clinically evident effects of fiber, starch and protein levels, as well as particle size, and also provide an explanation for the positive effects from time-based feed restriction in relation to gastrointestinal disease in fast growing rabbits. It should be noted that the integrated view presented in this paper primarily intends to explain the derailment as observed in rabbits with non-specific enteropathies. In practice, digestive problems could be due to various causes and could have various consequences (e.g., diarrhea, impaction). However, in the literature that was used for the building of our integrated view, it was not always specified what the exact observed digestive problems were, making it difficult to attribute the findings to a specific cause or type of digestive problem. As the conceptual pathways discussed in this paper describe quite general mechanisms, these pathways (or components thereof) may explain gastrointestinal derailment in a broader context, and resulting from many other and different causes. Nevertheless, it would be valuable to gain more insight into the histopathological and functional changes that occur in the various parts of the gastrointestinal tract with specific digestive problems to obtain an understanding of the disease mechanisms and how certain interventions can help to prevent or treat disease. In this respect, (changes in) the morphology or functioning of the *fusus coli* would be of particular interest to study further in both healthy and diseased animals, given the central role this structure plays in regulating gastrointestinal motility.

The framework as presented here also emphasizes the interconnectedness of the different components in the pathways, whereby any and all deviations from the optimal situation, whether in feed formulation or in the amount or ways in which the food is provided to rabbits, can lead to gastrointestinal derailment. The importance of providing adequately balanced diets with appropriate levels of fiber, starch and protein for optimal functioning of the gastrointestinal tract in (production) rabbits, as emphasized in our integrated model, has long been established in the literature. However, our model, and in particular the “chymus jam” pathway, also provides a place for and emphasizes the relevance of interventions focused at feed distribution across the day and fiber particle size to help avoid the cascade of effects that eventually lead to gastrointestinal derailment and digestive problems in meat production rabbits. While some initial empirical evidence already exists to support the hypothesized effects, it would be important to conduct further research to identify whether (1) temporal feed restriction reduces the incidence of non-specific enteropathies through preventing or resolving a potential chymus jam that may develop; and (2) providing diets that contain (higher percentages of) larger sized fiber particles reduces the incidence of non-specific enteropathies by optimizing gastrointestinal motility and functioning of the *fusus coli*. While the latter of the two hypotheses is conceptually sound and aligns with previous findings during studies on rabbit gastrointestinal physiology, it does still require explicit empirical support to determine its value in relation to the non-specific enteropathies, and determine what percentages of large fibers are helpful to avoid these problems. Aforementioned insights will be instrumental to help reconsider the appropriate dietary strategy and composition to optimize the balance between growth and production on the one hand and gastrointestinal health on the other. In addition, insight into the mechanisms through which the *fusus coli* affects (dys)functioning of the rabbit gastrointestinal tract, and factors that can help improve or restore its functioning (such as temporary fasting), will be helpful to offer new and more effective options to prevent and treat non-specific enteropathies and associated losses in production rabbits.

## 5 Conclusions

In this conceptual approach, which is supported by views in the literature [e.g., (12, 41, 49)] as well as empirical evidence [e.g., (15, 18, 42, 50, 51)], we have tried to provide an integrated view of how the gastrointestinal tract of (meat production) rabbits might derail under certain circumstances. Even though some uncertainties remain, it is likely that fiber deficiencies and excess amounts of easily fermentable substrates (i.e., starch and protein) both feed into the same process through mechanisms leading to “overload” and/or “chymus jam” at the level of the colon and caecum. The hypothesized mechanisms of derailment suggest a pivotal role of the *fusus coli* and fiber particle size in the derailment of the gastrointestinal tract under non-specific enteropathies. The framework provided here could serve as a starting point for further understanding the gastrointestinal problems in rabbits, thereby aiding in the development of strategies to avoid or reduce the occurrence of digestive problems in production rabbits and helping to improve their overall gastrointestinal health.

## Data availability statement

The original contributions presented in the study are included in the article/supplementary material, further inquiries can be directed to the corresponding author.

## Author contributions

MS: Conceptualization, Visualization, Writing—original draft. YZ: Writing—review & editing. KG: Conceptualization, Writing—review & editing.

## Footnotes

^1^Increase in ileal starch for the diets with higher starch levels was statistically significant.
